# Virulence profiling of Shiga toxin-producing *Escherichia coli* recovered from domestic farm animals in Northwestern Mexico

**DOI:** 10.3389/fcimb.2014.00007

**Published:** 2014-01-31

**Authors:** Bianca A. Amézquita-López, Beatriz Quiñones, Bertram G. Lee, Cristóbal Chaidez

**Affiliations:** ^1^Centro de Investigación en Alimentación y DesarrolloCuliacán, Mexico; ^2^Produce Safety and Microbiology Research Unit, U.S. Department of Agriculture/Agricultural Research Service, Western Regional Research CenterAlbany, CA, USA

**Keywords:** *Escherichia coli*, STEC, virulence genes, Shiga toxin, Vero cells, Mexico, zoonosis, foodborne pathogen

## Abstract

Shiga toxin-producing *Escherichia coli* (STEC) is a zoonotic enteric pathogen that causes human gastrointestinal illnesses. The present study characterized the virulence profiles of O157 and non-O157 STEC strains, recovered from domestic animals in small rural farms within the agricultural Culiacan Valley in Mexico. Virulence genes coding for adhesins, cytotoxins, proteases, subtypes of Shiga toxin (Stx), and other effectors were identified in the STEC strains by PCR. The genotyping analysis revealed the presence of the effectors *nleA*, *nleB*, *nleE*, and *nleH1-2*, *espK*, and *espN* in the O157:H7 and O111:H8 STEC strains. Furthermore, the genes encoding the autoagglutinating adhesin (Saa) and subtilase (SubA) were exclusively identified in the O8:H19 *eae*-negative strains. The adhesin (*iha*) and the silent hemolysin (*sheA*) genes were detected in 79% of the O157 and non-O157 strains. To examine the relative toxicities of the STEC strains, a fluorescent Vero cell line, Vero-d2EGFPs, was employed to measure the inhibition of protein synthesis by Stx. Analysis of culture supernatants from serotype O8:H19 strains with the *stx* gene profile *stx*_1a_, *stx*_2a_, and *stx*_2c_ and serotypes O75:H8 and O146:H8 strains with the *stx* gene profile *stx*_1a_, *stx*_1c_, and *stx*_2b_, resulted in a significant reduction in the Vero-d2EGFP fluorescent signal. These observations suggest that these non-O157 strains may have an enhanced ability to inhibit protein synthesis in Vero cells. Interestingly, analysis of the *stx*_2c_-positive O157:H7 strains resulted in a high fluorescent signal, indicating a reduced toxicity in the Vero-d2EGFP cells. These findings indicate that the O157 and non-O157 STEC strains, recovered in the Culiacan Valley, display distinct virulence profiles and relative toxicities in mammalian cells and have provided information for evaluating risks associated with zoonotic STEC in this agricultural region in Mexico.

## Introduction

Shiga toxin-producing *Escherichia coli* (STEC) is considered to be a major cause of foodborne disease and can cause a wide variety of disease symptoms in humans, ranging from watery and bloody diarrhea to the life-threatening diseases such as hemorrhagic colitis, and hemolytic uremic syndrome (HUS) (Tarr et al., 2005; Gyles, 2007; Karmali et al., 2010; Scallan et al., 2011; Melton-Celsa et al., 2012). Cattle are considered to be the major carrier of STEC strains (Gyles, 2007; Ferens and Hovde, 2011). However, additional studies that examined important animal reservoirs for these bacterial pathogens have indicated that small domestic ruminants, including sheep and goats, have been implicated as carriers of STEC (Ogden et al., 2005; Gyles, 2007; La Ragione et al., 2009; Ferens and Hovde, 2011; Mandrell, 2011). Furthermore, STEC strains have been also detected in other domestic and wild animals, including cats, dogs, rodents, deer, birds, feral pigs, chickens, and insects (Cooley et al., 2007; Ferens and Hovde, 2011; Mandrell, 2011).

Severe disease in humans has been associated with more than 100 serotypes of STEC (Gould et al., 2009; Mathusa et al., 2010). Serotype O157:H7 is responsible for most outbreaks in the United States (Karmali, 2009; Hoefer et al., 2011; Melton-Celsa et al., 2012). Additional epidemiological studies have indicated that six non-O157 serogroups, O26, O45, O103, O111, O121, and O145, have been associated with severe disease symptoms in North America (Johnson et al., 2006; Gould et al., 2009; Stigi et al., 2012). Additionally, STEC of serogroups, O91, O104, O113, and O128 have been reported to be significant causes of human infections worldwide (Brooks et al., 2005; Bettelheim, 2007; Mathusa et al., 2010; Beutin and Martin, 2012). Thus, these findings have indicated that strains with certain non-O157 serogroups may be potentially as virulent as strains with the O157:H7 serotype (Bettelheim, 2007; Coombes et al., 2011; Beutin and Martin, 2012; Stigi et al., 2012).

The production of Shiga toxins (Stx) by STEC contributes to the development of the life-threatening disease symptoms in humans (Karmali et al., 1983; Karmali, 1989). The Stx family has been categorized into two major types, Stx1 and Stx2. In particular, distinct subtypes of Stx1, Stx1a, Stx1c and Stx1d, have been identified (Scheutz and Strockbine, 2005; Scheutz et al., 2012). By contrast, the Stx2 group consists of a heterogeneous and diverse group of subtypes, and seven subtypes of Stx2, corresponding to Stx2a, Stx2b, Stx2c, Stx2d, Stx2e, Stx2f, and Stx2g, have been documented (Scheutz and Strockbine, 2005; Scheutz et al., 2012). Epidemiological and molecular genotyping studies of STEC have demonstrated that there is a strong correlation between strains with certain *stx*_2_ subtypes and severe illness such as bloody diarrhea and HUS. STEC strains positive for the subtypes *stx*_2*a*_, *stx*_2*c*_, or *stx*_2*d*_ were found to be associated with an enhanced virulence and with the development of the HUS in humans (Friedrich et al., 2002; Beutin et al., 2004; Bielaszewska et al., 2006; Persson et al., 2007). Other subtypes of Stx1 and Stx2 appear to be associated with mild disease or asymptomatic carriage (Friedrich et al., 2002, 2003; Beutin et al., 2004; Bielaszewska et al., 2006; Scheutz et al., 2012).

Many STEC strains that produce Stx do not cause HUS, demonstrating that additional virulence factors may be required to cause illness in humans (Bolton, 2011). For example, virulence factors present on pathogenicity islands, such as the locus of enterocyte effacement (LEE) and the non-LEE effectors, have been implicated in host colonization and disease (Bettelheim, 2007; Bolton, 2011; Coombes et al., 2011). In particular, a key virulence factor responsible for the attachment to intestinal epithelial cells is the LEE-encoded *eae* gene (Jerse et al., 1990; Kaper, 1998). An additional adhesin, Iha, the iron-regulated gene A homolog adhesin, may contribute to the attachment of LEE-positive and LEE-negative strains (Tarr et al., 2000; Schmidt et al., 2001). Moreover, the Nle effectors, not encoded by the LEE region, are proposed to be involved in altering the host cell response and have been linked to the disease severity associated with non-O157 STEC (Coombes et al., 2008; Karmali et al., 2010; Melton-Celsa et al., 2012). Other chromosomal and plasmid virulence genes, encoding proteases (*espP*), cytotoxins (*subA*), and adhesins (*saa*), may contribute to STEC pathogenesis by allowing bacterial attachment and colonization of the human epithelium (Karmali et al., 2010; Bolton, 2011; Melton-Celsa et al., 2012). The detection of these virulence genes in STEC strains would provide key information for the identification of risk factors that may potentially contribute to the development of human disease.

In the present study, a molecular characterization study was conducted to further examine the virulence potential of STEC strains, previously recovered from feces of domestic animals in small rural farms within the agricultural Culiacan Valley in Northwestern Mexico (Amézquita-López et al., 2012). The small rural farms were located in communities where the primary purpose of raising livestock is for local consumption (Amézquita-López et al., 2012). To determine the virulence profiles of STEC strains from animal reservoirs in this agricultural region in Mexico, the present study identified the *stx* subtypes as well as several virulence factors that have been associated with pathogenic STEC strains. The activity of the Stx subtypes, expressed by the tested STEC strains, was also further examined to obtain more detailed information on their ability to inhibit protein synthesis in mammalian cells.

## Materials and methods

### Bacterial strains and growth conditions

A subset of 29 STEC strains, previously recovered from various animal reservoirs in the Culiacan Valley in Mexico (Amézquita-López et al., 2012), was studied (Table 1). The subset of strains was selected based on serotype and on the recovery from distinct dates, sampling sites and animal reservoirs (Amézquita-López et al., 2012).

**Table 1:** **List of *E. coli* O157 and non-O157 strains analyzed in this study**.

**Strain**	**Serotype^a^**	**Sampling date**	**Source**	**Region^b^**
RM8744	O157:H7	18-Nov-08	Cattle	Iraguato
RM8745	O73:H4	02-Dec-08	Sheep	El Castillo
RM8747	O15:NT	22-Jul-08	Cattle	Agua Caliente
RM8748	O73:NT	22-Jul-08	Cattle	Agua Caliente
RM8749	O20:H4	12-Aug-08	Sheep	El Castillo
RM8752	O75:H8	07-Oct-08	Sheep	Cofradia de Navolato
RM8753	O157:H7	02-Dec-08	Sheep	Cofradia de Navolato
RM8755	O111:H8	20-Jan-09	Sheep	Cofradia de Navolato
RM8756	O146:H21	20-Jan-09	Sheep	Jotagua
RM8759	O157:H7	20-Jan-09	Sheep	Cofradia de Navolato
RM8760	O75:H8	20-Jan-09	Sheep	Cofradia de Navolato
RM8761	O146:H21	20-Jan-09	Sheep	El Castillo
RM8762	O146:H8	20-Jan-09	Sheep	El Castillo
RM8763	O75:H8	03-Feb-09	Sheep	Cofradia de Navolato
RM8768	O157:H7	20-Jan-09	Cattle	Cofradia de Navolato
RM8772	O8:H19	17-Feb-09	Cattle	El Castillo
RM8776	O8:H19	03-Feb-09	Cattle	Iraguato
RM8778	O75:H8	25-Feb-09	Sheep	Cofradia de Navolato
RM8781	O157:H7	25-Feb-09	Sheep	Cofradia de Navolato
RM8922	O157:H7	10-Mar-09	Cattle	Cofradia de Navolato
RM8923	O75:H8	10-Mar-09	Cattle	Cofradia de Navolato
RM8928	O157:H4	10-Mar-09	Cattle	El Castillo
RM8929	O75:H8	10-Mar-09	Sheep	Cofradia de Navolato
RM9450	O157:H7	10-Mar-09	Sheep	Cofradia de Navolato
RM9452	O157:H7	26-May-09	Sheep	Cofradia de Navolato
RM9454	O157:H7	26-May-09	Cattle	Cofradia de Navolato
RM9456	O157:H7	07-Apr-09	Cattle	Cofradia de Navolato
RM9458	O157:H7	24-Mar-09	Chicken	Agua Caliente
RM9462	O157:H7	03-Jun-09	Cattle	Iraguato
RM13865	O75:H8	07-Apr-09	Cattle	Cofradia de Navolato

a *NT, Non-typeable H-antigen*.

b *Sampling sites correspond to regions in the Culiacan Valley, Sinaloa, Mexico (Amézquita-López et al., 2012)*.

The method employed to isolate the STEC strains (Table 1) from fecal samples from various domestic animals was previously described (Amézquita-López et al., 2012). The characteristics and sources of the STEC reference strains that were used in this study are described in Table 2. Bacteria were routinely propagated under aerobic conditions at 37°C on Luria-Bertani (LB) agar (Difco, Detroit, MI).

**Table 2:** **Shiga toxin-producing *Escherichia coli* reference strains used in this study**.

**Strain**	**Other strain designations**	**Serotype**	**Source**	**Location**	**Provider or reference^a^**
RM2084	EDL-933; DEC 4f	O157:H7	Meat	United States	ECRC (Reid et al., 1999)
RM7004	E32511; TW02883	O157:H	Human	United States	The STEC Center (Schmitt et al., 1991)
RM7005	EH250; TW081611	O118:H12	Human	Belgium	The STEC Center (Piérard et al., 1998)
RM7006	B2F1; TW01393	O91:H21	Human	Canada	The STEC Center (Ito et al., 1990)
RM7110	NADC2228; S1191	O139:NM	Pig	United States	Evelyn Dean-Nystrom (Weinstein et al., 1988)
RM7203	SC-0012	O168:H8	Coyote	United States	Michael B. Cooley (Cooley et al., 2013)
RM7369	SP-0082-G9	ONT:H7	Pig	United States	Michael B. Cooley (Cooley et al., 2013)
RM7508	MA146B-A7	O128:H2	Deer	United States	Michael B. Cooley (Cooley et al., 2013)
RM7519	F260-H2	O113:H21	Cattle	United States	Michael B. Cooley (Cooley et al., 2013)

a *Contact information of strain providers: ECRC, E. coli Reference Center, College of Agricultural Sciences, The Pennsylvania State University, University Park, PA, USA; The STEC Center, National Food Safety and Toxicology Center, Michigan State University, East Lansing, MI, USA; Michael B. Cooley, USDA/ARS, Western, Regional Research Center, Produce Safety and Microbiology Research Unit, Albany, CA, USA; Evelyn Dean-Nystrom, Iowa State University, National Animal Disease Center, Veterinary Microbiology and Preventive Medicine, Ames, Iowa, USA*.

### Polymerase chain reaction for amplification of *stx* subtypes and other virulence genes

For the detection of *stx* subtypes and other virulence genes, the following STEC reference strains (Table 2) were used as a control for the PCR amplification of *ent/espL2*, *espK*, *espN*, *espP*, *etpD*, *ihA*, *katP*, *nleA*, *nleB*, *nleE*, *nleH1-2*, *sheA*, *stx*_1a_, and *stx*_2a_ (RM2084); *stx*_2c_ (RM7004); *stx*_2b_ (RM7005); *saa* and *stx*_2d_ (RM7006); *stx*_2f_ (RM7007); *stx*_2e_ (RM7110); *stx*_2g_ (RM7203); *stx*_1d_ (RM7369); *stx*_1c_ (RM7508); *hlyA* (RM10227). All PCR amplifications were performed by using primers as shown in Table 3. As template for the PCR reaction, cultures of the STEC strains were grown aerobically in tryptic soy broth (Beckton Dickinson, Sparks, MD) for 24 h with constant shaking (200 rpm) at 37°C, and 100 μL of the bacterial cultures were collected by centrifugation at 2000 ×*g* for 5 min. Cell pellets were resuspended in 100 μl of HyPure™ molecular biology-grade water (HyClone Laboratories, Inc., Logan, UT) and incubated at 95°C for 20 min, as in previous studies (Quiñones et al., 2011, 2012). The lysates were centrifuged at 2000 ×*g* for 5 min, and the supernatants were collected and frozen until further use. The PCR amplifications consisted of a 25 μl reaction mixture, each containing 5 μL of the bacterial crude lysate, 0.5 μM of each primer (Eurofins MWG Operon, Huntsville, AL), and 12.5 μl of 2× GoTaq® Green Master Mix (Promega Corporation, Madison, WI). The reaction mixtures were placed in a Dyad Peltier Thermal Cycler (Bio-Rad Laboratories, Hercules, CA), as in previous studies (Amézquita-López et al., 2012). The virulence genes were amplified with PCR cycling conditions, as described in the references listed in Table 3. Amplified products were analyzed in 2% agarose gels containing 0.04 μl/ml GelRed Nucleic Acid Stain (Phenix Research, Candler, NC).

**Table 3:** **List of DNA oligonucleotides used in this study for PCR amplification**.

**Target gene^a^**	**Forward sequence (5′ → 3′)**	**Reverse sequence (5′ → 3′)**	**Amplicon size (bp)**	**Reference**
*ent/espL2*	CACATCATTAGAAGTTCATT	AGTCCTGCTCCCATAGCAAA	342	Quiñones et al., 2012
*espK*	GTAGCGCCACAGACAGCATA	ATCAGGCATCCCTTCAACAC	242	Kyle et al., 2012
*espN*	TTTCTTTCGTGACGCTGATG	GCACCGGAGAATCATCGTAT	155	Kyle et al., 2012
*espP*	GCACTGTCTGGCGGTGAATA	CGTCCAGATTCCCGTTTATG	202	Quiñones et al., 2012
*etpD*	TTGGATGACGGCGAAACTG	AGATGATACGCTGTTGGGAG	85	Bugarel et al., 2010b
*hlyA*	GTCTGCAAAGCAATCCGCTGCAAATAAA	CTGTGTCCACGAGTTGGTTGATTAG	561	Kerényi et al., 2005
*iha*	GTGATGATTGTCTCGGCATC	GTAACTGGCTGGCATTCCWC	207	Kyle et al., 2012
*katP*	GCGGAAGAGAAGATGACTGG	GCACCATGTGCTTTACCAAA	277	Quiñones et al., 2012
*nleA*	TGGATTAACDGCTCARGTDGTTCG	GCATTGGTAAGYARGGCATA	267	Kyle et al., 2012
*nleB*	GGAAGTTTGTTTACAGAGACG	AAAATGCCGCTTGATACC	297	Coombes et al., 2008
*nleE*	GTATAACCAGAGGAGTAGC	GATCTTACAACAAATGTCC	260	Coombes et al., 2008
*nleH1-2*	GCCTGATAATCGTGTTTTATC	CGCATAATCCACTGGAGGTAA	295	Kyle et al., 2012
*saa*	CCAATCAACAGTTTCGTCAA	GCAATAGCCTGTTCATCACG	166	Quiñones et al., 2012
*sheA*	GAGGCGAATGATTATGACTG	ACTTCAGGTACCTCAAAGAG	920	Kerényi et al., 2005
*stx*_1a_	CACGTTACAGCGTGTTGCA	CGCCCACTGAGATCATCC	219	Kyle et al., 2012
*stx*_1c_	GAACGAAATAATTTATATGT	CTCATTAGGTACAATTCT	555	Koch et al., 2001
*stx*_1d_	CTTTTCAGTTAATGCGATTGCT	AACCCCATGATATCGACTGC	192	Bürk et al., 2003
*stx*_2a_	AGATATCGACCCCTCTTGAA	GTCAACCTTCACTGTAAATG	969	Nakao et al., 2002
*stx*_2b_	TATACGATGACACCGGAAGAAG	CCTGCGATTCAGAAAAGCAGC	300	Nakao et al., 2002
*stx*_2c_	TTTTATATACAACGGGTA	GGCCACTTTTACTGTGAATGTA	163	Nakao et al., 2002; Zheng et al., 2008
*stx*_2d_	CTTTATATACAACGGGTG	CTGAATTGTGACACAGATTAC	359	Zheng et al., 2008
*stx*_2e_	CAGGAAGTTATATTTCCGTAGG	GTATTCTCTTCCTGACACCTTC	911	Nakao et al., 2002
*stx*_2f_	TTTACTGTGGATTTCTCTTCGC	TCAGTAAGATCCTGAGGCTTG	875	Nakao et al., 2002
*stx*_2g_	GTTATATTTCTGTGGATATC	GAATAACCGCTACAGTA	573	Leung et al., 2003
*subA*	CGGCTTATCATCCTGTCAGC	TATAGCTGTTGCTTCTGACG	233	Quiñones et al., 2012

a The stx_1_ and stx_2_ subtypes are listed with new stx nomenclature, as recently described (Feng et al., 2011; Scheutz et al., 2012)

### Vero cell-based method to detect STX activity

The Stx activity of the STEC strains was measured using a Vero cell line, Vero-d2EGFP, that harbored a destabilized variant (*t*_1/2_ = 2 h) of the enhanced green fluorescent protein (EGFP) (Quiñones et al., 2009; Quiñones and Swimley, 2011). To monitor the Stx-induced inhibition of protein synthesis, the tested STEC strains (Table 1), the Stx-expressing O157:H7 strain RM2084 (positive control) (Table 2), and the Stx-negative O157:H4 strain RM8928 (Amézquita-López et al., 2012) (negative control) were inoculated in 1 ml of sterile LB broth (Difco, Detroit, MI). All *E. coli* strains were grown aerobically for 24 h at 37°C with shaking at 200 rpm and were then centrifuged at 2000 ×*g* for 15 min. The culture supernatants were filter-sterilized using 0.45 μm polyvinylidene fluoride syringe filters (Durapore® membranes, Millipore Corporation, Billerica, MA) and were frozen at −20°C until further use (Quiñones and Swimley, 2011). One day prior to intoxication, the Vero-d2EGFP cells were seeded at 10,000 cells per well in Greiner black 96-well microplates with clear bottoms (VWR International, Aurora, CO) and were grown at 5% CO_2_ and 37°C under humidified conditions in Ham's F-12 complete medium, supplemented with 10% fetal bovine serum and 1% penicillin-streptomycin (Gibco BRL, Grand Island, NY) (Quiñones et al., 2009; Quiñones and Swimley, 2011). The Vero-d2EGFP cells were then exposed to Ham's F-12 complete medium containing a tenfold dilution of the cell-free supernatants from each strain and were incubated for 16 h at 37°C in a 5% CO_2_ humidified incubator. The EGFP fluorescence from the Vero-d2EGFP cells was measured using a Synergy HT Multi-Detection Microplate Reader (BioTek, Winooski, VT) with the 485/20 nm excitation filter and the 528/20 nm emission filter (Quiñones et al., 2009; Quiñones and Swimley, 2011). All measurements were performed in triplicate, and the results were expressed as percentages of the fluorescence values obtained for culture supernatant-treated Vero-d2EGFP cells when compared to the fluorescence values from control Vero-d2EGFP cells incubated without culture supernatants. To determine statistical differences in the Stx activity among the STEC strains, the results were analyzed by performing a *k*-means clustering using the Hartigan and Wong algorithm (Hartigan and Wong, 1979) with the R Statistical Software (version 3.0.1; R Foundation for Statistical Computing, Vienna, Austria) (R-Core Team, 2013). The distinct clusters were further validated by measuring the Dunn Index with the *clValid* R Package (Brock et al., 2008).

## Results

### Virulence gene profiles of STEC strains from domestic farm animals

To further characterize the virulence potential of STEC strains recovered from domestic farm animals in the agricultural Culiacan Valley region in Mexico, the presence of genes, associated with pathogenic STEC strains, was identified. Our initial analysis focused on the identification of the subtypes of Stx, a virulence factor that has been attributed to the development of serious disease symptoms in humans (Karmali et al., 2010; Bolton, 2011; Melton-Celsa et al., 2012). The results indicated that 97% (28/29) of the O157 and non-O157 STEC strains, recovered from sheep, cattle and chickens, were PCR-positive for genes encoding *stx*_2_ subtypes (Table 4). By contrast, *stx*_1_ subtypes were only identified in the non-O157 strains from sheep and cattle. Interestingly, the *stx*_2c_ subtype was detected in 51% (15/29) of the strains selected from the different animal sources and was predominantly identified in strains with the O157:H7 serotype (Table 4). Furthermore, the *stx*_2d_ subtype was only identified in the ovine strain RM8749 belonging to serotype O20:H4. Our results also demonstrated that 45% (13/29) of the recovered STEC strains were positive for more than one gene encoding *stx* subtypes in the same strain. In particular, strains with serotypes O73:H4, O75:H8, O146:H8, and O146:H21 were found to harbor the *stx* gene profile *stx*_1a_, *stx*_1c_, and *stx*_2b_ (Table 4). Moreover, the *stx* gene profile *stx*_1a_, *stx*_2a_, and *stx*_2c_ was exclusively found in the O8:H19 strains recovered from cattle. None of the STEC strains recovered from domestic animals in rural farms in the Culiacan Valley were PCR-positive for the *stx* subtypes *stx*_1d_, *stx*_2e_, *stx*_2f_, or *stx*_2g_.

**Table 4:** **Identification of virulence genes in *E. coli* O157 and non-O157 strains used in this study**.

**Serotype**	**Strain**	**Source**	**Virulence profile**
O8:H19	RM8772	Cattle	*espP, saa, stx*_1a_*, stx*_2a_*, stx*_2c_*, subA*
	RM8776	Cattle	
O15:NT	RM8747	Cattle	*ent/espL2, espP, katP, stx*_2c_
O20:H4	RM8749	Sheep	*stx*_2d_
O73:NT	RM8748	Cattle	*ent/espL2, espP, katP, stx*_2a_
O73:H4	RM8745	Sheep	*stx*_1a_*, stx*_1c_*, stx*_2b_
O75:H8	RM8752	Sheep	*iha, sheA, stx*_1a_*, stx*_1c_*, stx*_2b_
	RM8760	Sheep	
	RM8763	Sheep	
	RM8778	Sheep	
	RM8923	Cattle	
	RM8929	Sheep	
	RM13865	Cattle	
O111:H8	RM8755	Sheep	*ent/espL2, espK, espN, iha, nleA, nleB, nleE, nleH1-2, sheA, stx*_1a_
O146:H8	RM8762	Sheep	*iha, sheA, stx*_1a_*, stx*_1c_*, stx*_2b_
O146:H21	RM8756	Sheep	*iha, sheA, stx*_1a_, *stx*_1c_, *stx*_2b_
	RM8761	Sheep	
O157:H7	RM8744	Cattle	*ent/espL2, espK, espN, espP, etpD, iha, katP, nleA, nleB, nleE, nleH1-2, sheA, stx*_2c_
	RM8753	Sheep	
	RM8759	Sheep	
	RM8768	Cattle	
	RM8781	Sheep	
	RM8922	Cattle	
	RM9450	Sheep	
	RM9452	Sheep	
	RM9454	Cattle	
	RM9456	Cattle	
	RM9458	Chicken	
	RM9462	Cattle	

Given that Stx is not the only virulence determinant that is responsible for full pathogenicity (Karmali et al., 2010; Bolton, 2011; Melton-Celsa et al., 2012), the STEC strains isolated from this agricultural region were further screened for the presence of additional markers encoding adhesins, cytotoxins, proteases, and other effectors. The virulence typing analysis revealed the presence of the non-LEE encoded effectors, *nleA*, *nleB*, *nleE*, and *nleH1-2*, in the recovered O157:H7 strains from sheep, cattle, and chicken as well as in the recovered O111:H8 strains from sheep (Table 4). Other effectors, *espK*, and *espN*, were also identified in the O157:H7 and O111:H8 strains. However, the plasmid-encoded *etpD* gene was only detected in the O157:H7 strains. Furthermore, *saa* and *subA* genes were exclusively identified in the O8:H19 cattle strains (Table 4). Finally, the iron-regulated adhesion gene (*iha*) and the cytolysin A gene (*sheA*) were both present in 79% (23/29) of the non-O157 and O157 strains. The ovine strains belonging to serotypes O20:H4 and O73:H4 were found to be negative for the presence of the accessory virulence determinants that were tested in the present study (Table 4).

### STX activity in STEC strains isolated from domestic farm animals

**Figure 1:**
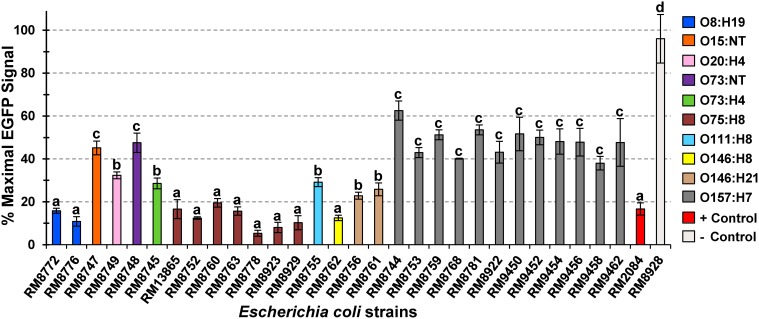
**Effect of *Escherichia coli* O157 and non-O157 culture supernatants on Vero-d2EGFP fluorescence**. Vero-d2EGFP cells were incubated in Ham's F-12 complete medium containing tenfold dilutions of cell-free culture supernatants from the tested *Escherichia coli* O157 and non-O157 strains (Table 1). Cell-free culture supernatants from the Stx-producing *E. coli* O157:H7 strain RM2084 and from the non-Stx producing *E. coli* O157:H4 strain RM8928 were used as positive and negative controls, respectively. The average ± standard deviation of three independent experiments with duplicate samples for each strain is shown. Bars with the same lowercase letter represent significantly distinct clusters according to the *k*-means clustering algorithm and the *clValid* R Software package (Brock et al., 2008; R-Core Team, 2013).

A quantitative and sensitive cell-based assay was further employed to examine the activity of the Stxs expressed by the O157 and non-O157 strains recovered from domestic animals in the Culiacan Valley. A Vero cell line, Vero-d2EGFP, was employed to measure the inhibition of protein synthesis by Stx in mammalian cells (Quiñones et al., 2009; Quiñones and Swimley, 2011). Consequently, incubation with active Stx results in a reduction of the EGFP fluorescent signal that is detected from the Vero-d2EGFP cells (Quiñones et al., 2009; Quiñones and Swimley, 2011). Our results indicated low levels of fluorescence, ranging from 5.4 to 19.5%, were observed when the Vero-d2EGFP cells were incubated with culture supernatants from several STEC strains with the serotypes O8:H19, O75:H8, and O146:H8 (Figure 1), recovered from sheep and cattle. Further statistical analysis of the detected EGFP fluorescence indicated that these STEC strains with serotypes O8:H19, O75:H8 and O146:H8 belong to the same *k*-means cluster, suggesting that the Stx expressed by these non-O157 strains had similar effects on the EGFP fluorescence. Moreover, significantly reduced levels of EGFP fluorescence to approximately 16.7% were also observed after incubation with cell-free culture supernatants from the positive control O157:H7 strain RM2084 (Figure 1).

Intermediate levels of EGFP fluorescence, ranging from 22.9 to 32.4% were observed after incubation with culture supernatants from the ovine STEC strains belonging to serotypes O20:H4, O73:H4, O111:H8, and O146:H21. Interestingly, analysis of the culture supernatants from all O157:H7strains, recovered from sheep, cattle and chickens in the Culiacan Valley, resulted in significantly higher levels of EGFP fluorescence (Figure 1). The levels of EGFP fluorescence when testing the supernatants from the O157:H7 strains in the Vero-cell based assay ranged from 38.0 to 62.6%. High levels of EGFP fluorescence that also belong to the same *k*-means cluster group as the O157:H7 strains were also observed when testing culture supernatants from the O15:NT and O73:NT strains (Figure 1). No reduction of EGFP fluorescence, averaging 98% detected fluorescence, was observed after incubation with culture supernatants from the negative control O157:H4 strain RM8928 strain lacking an *stx* gene (Figure 1). The fluorescence in the Vero-d2EGFP cells still remained when testing culture supernatants from an *stx*-negative O157:H7 strain RM4876 (Quiñones et al., 2009) or after incubation with bacterial growth media without toxin added (data not shown).

## Discussion

In the present study, a genotyping and functional analysis was conducted to further characterize STEC strains, recovered from domestic animals in rural farms in the Culiacan Valley, which is considered one of the most important agricultural regions in Mexico (Amézquita-López et al., 2012). Given that the rural farms were located in communities that may not follow efficient management of animal wastes (Jiménez et al., 2011; Amézquita-López et al., 2012), an understanding of the virulence potential of the STEC strains recovered from animal reservoirs in this agricultural region would assist in the development of control measures to prevent the dispersal and transmission of pathogens throughout the environment that could lead to human infections associated with STEC.

The virulence typing analysis revealed that all O157:H7 and O111:H8 STEC strains from domestic animal reservoirs in the Culiacan Valley were positive for several of the *nle* genes, known to be located in the genomic islands OI-122 and OI-71 (Coombes et al., 2008). A previous study showed that these O157 and O111 STEC strains were also positive for *eae* (Amézquita-López et al., 2012). Thus, these findings revealed that these STEC strains from the Culiacan Valley harbor the gene signature, *eae*, *ent/espL2*, *nleA*, *nleB*, *nleF*, and *nleH1-2*, which has been proposed to be present in STEC strains with high virulence for humans (Bugarel et al., 2010a). Moreover, EtpD, the pO157 plasmid-encoded type II secretory pathway protein (Burland et al., 1998), was exclusively identified in O157:H7 strains. Finally, the STEC autoagglutinating adhesin (Saa) and subtilase cytotoxin (SubA) were specifically detected in the O8:H19 cattle strains, previously shown to be *eae*-negative and to display a limited genomic diversity by multiple-locus variable-number tandem repeat analysis (Amézquita-López et al., 2012). The findings from the present study are in agreement with other reports that documented Saa and SubA to be associated with non-O157 LEE-negative strains (Paton et al., 2001; Jenkins et al., 2003; Kumar et al., 2004; Toma et al., 2004; Zweifel et al., 2004; Kobayashi et al., 2013).

Approximately 79% of the O157 and non-O157 recovered STEC harbored both the *iha* and *sheA* genes. Previous studies demonstrated that *iha*, which codes for the iron-regulated gene A homolog adhesin, has been commonly observed in both LEE-positive as well as LEE-negative strains with different serotypes (Tarr et al., 2000; Schmidt et al., 2001). Moreover, *sheA*, encoding the cytolysin A or “silent hemolysin” has been shown to be prevalent in certain non-pathogenic *E coli* strains and in other enteropathogenic *E. coli* strains (Del Castillo et al., 1997; Fernández et al., 1998; Ludwig et al., 2004). All O157:H7 strains were positive for *katP*, a gene mostly identified in STEC strains belonging to seropathotypes associated with HUS (Bugarel et al., 2010b, 2011; Kobayashi et al., 2013). However, the present study also detected *katP* in O73:NT and O15:NT strains, belonging to serogroups not implicated in causing any human illness (Hussein, 2007).

To examine the relative toxicities of Stx subtypes expressed by the recovered STEC strains, the Vero-d2EGFP fluorescent assay was employed. The assay uses the Vero-d2EGFP cell line, expressing a destabilized variant of EGFP (Quiñones et al., 2009), and measures in mammalian cells the inhibition of protein synthesis by Stx (Quiñones et al., 2009; Quiñones and Swimley, 2011). Given that Vero cells are highly responsive to the effects of Stx (Keusch et al., 1995), the Vero-d2EGFP fluorescent assay is thus a sensitive and quantitative method to examine the potential relative toxicities of STEC strains. The results from the present study demonstrated that STEC strains with serotypes O8:H19, O75:H8, and O146:H8, serotypes previously associated with severe disease in humans (Boerlin et al., 1999; Blanco et al., 2003; Hussein, 2007), displayed a significant reduction in the EGFP signal from the Vero-d2EGFP cells to similar levels as the positive control O157:H7 strain RM2084. Moreover, the molecular typing study revealed that the *stx* gene profile *stx*_1a_, *stx*_2a_, and *stx*_2c_ was exclusively found in the O8:H19 cattle strains while the *stx*_1a_, *stx*_1c_, and *stx*_2b_ profile was detected in the O73:H4, O75:H8, O146:H8 and O146:H21 strains from cattle and sheep. In summary, these findings have indicated that these non-O157 strains, possessing multiple *stx* subtypes, appear to be more efficient at inhibiting protein synthesis in mammalian cells.

Interestingly, analysis of the Stx activity from the *stx*_2c_-positive O157:H7 strains, recovered from chicken, cattle, and sheep in the Culiacan Valley, indicated that the Vero-d2EGFP fluorescence levels were on average threefold higher when compared to the positive control O157:H7 strain RM2084. These findings suggested that the O157:H7 strains from this region in Mexico may have a significantly lower ability to inhibit protein synthesis in mammalian cells. Previous reports have documented that the Stx2a, Stx2c, and Stx2d subtypes have been associated with severe disease symptoms, including HUS and bloody diarrhea, as well as with differential toxicities in mammalian cells (Friedrich et al., 2002; Ethelberg et al., 2004; Persson et al., 2007; Manning et al., 2008; Müthing et al., 2009; Fuller et al., 2011; Quiñones and Swimley, 2011). However, recent evidence has indicated that purified Stx2c appears to have a reduced potency at inhibiting protein synthesis and metabolic activity in mammalian cells and a lower toxicity in mice (Fuller et al., 2011). The findings from the present study have demonstrated that the *stx*_2c_-positive O157:H7 strains from this region in Mexico, previously shown to be closely-related by multiple-locus variable-number tandem repeat analysis (Amézquita-López et al., 2012), were less toxic to Vero cells although they possessed other key accessory virulence factors.

Previous reports have documented that the amounts of Stx2 produced may define the severity of disease caused by STEC strains (Zhang et al., 2000; Dean-Nystrom et al., 2003; Muniesa et al., 2004), and the differential expression and induction of Stx2 subtypes appears to contribute to the relative virulence of the STEC strain (Muniesa et al., 2004; Zhang et al., 2005; De Sablet et al., 2008). Therefore, future work is aimed at further characterizing the amounts of Stx produced after induction under different conditions to obtain a more detail understanding of the pathogenic potential of O157 and non-O157 STEC strains from diverse sampling sites and sampling sources in the agricultural Culiacan Valley region in Mexico.

### Conflict of interest statement

The authors declare that the research was conducted in the absence of any commercial or financial relationships that could be construed as a potential conflict of interest.
